# Development of VPC-70619, a Small-Molecule N-Myc Inhibitor as a Potential Therapy for Neuroendocrine Prostate Cancer

**DOI:** 10.3390/ijms23052588

**Published:** 2022-02-26

**Authors:** Anh-Tien Ton, Jane Foo, Kriti Singh, Joseph Lee, Anastasia Kalyta, Helene Morin, Carl Perez, Fuqiang Ban, Eric Leblanc, Nada Lallous, Artem Cherkasov

**Affiliations:** Vancouver Prostate Centre, University of British Columbia, 2660 Oak Street, Vancouver, BC V6H 3Z6, Canada; aton@prostatecentre.com (A.-T.T.); jfoo@prostatecentre.com (J.F.); sn.kriti@gmail.com (K.S.); jlee@prostatecentre.com (J.L.); akalyta@student.ubc.ca (A.K.); hmorin@prostatecentre.com (H.M.); cperez@virogin.com (C.P.); fban@prostatecentre.com (F.B.); eric.jj.leblanc@gmail.com (E.L.)

**Keywords:** prostate cancer, Myc, computer-aided drug design, therapeutic target, drug discovery

## Abstract

The Myc family of transcription factors are involved in the development and progression of numerous cancers, including prostate cancer (PCa). Under the pressure of androgen receptor (AR)-directed therapies resistance can occur, leading to the lethal form of PCa known as neuroendocrine prostate cancer (NEPC), characterized among other features by N-Myc overexpression. There are no clinically approved treatments for NEPC, translating into poor patient prognosis and survival. Therefore, there is a pressing need to develop novel therapeutic avenues to treat NEPC patients. In this study, we investigate the N-Myc-Max DNA binding domain (DBD) as a potential target for small molecule inhibitors and utilize computer-aided drug design (CADD) approaches to discover prospective hits. Through further exploration and optimization, a compound, VPC-70619, was identified with notable anti-N-Myc potency and strong antiproliferative activity against numerous N-Myc expressing cell lines, including those representing NEPC.

## 1. Introduction

The Myc protein family are transcription factors regulating communication networks within cells by binding to DNA and modulating the expression of numerous genes involved in cell growth and division. Dysregulation of Myc, especially N-Myc, is strongly associated with the development of the most resistant malignancies, including neuroendocrine prostate cancer (NEPC) [1,2,3,4] for which efficient therapeutic options are limited [5,6]. Hence, development of novel molecules targeting N-Myc onco-driver has a great potential as therapeutic intervention for NEPC [7].

Myc has always been considered a difficult target and often classified as undruggable, due to its intrinsically disordered nature and the lack of distinct binding pockets on its surface [8,9,10,11]. In addition, directly blocking Myc transcription might trigger unwanted side effects because of its central role in controlling gene expression in cells [12]. Deployment of cutting-edge computer-aided drug design (CADD) approaches and refined experimental methodologies have been critical in recent years to overcome the challenges of targeting Myc with small molecules [13,14,15]. Novel strategies were recently developed to inhibit Myc-driven effects by disrupting the Myc-Max heterodimer, or by interfering with the formation of the Myc-Max-DNA complex.

For example, compound EN4 was recently designed as a covalent ligand targeting the Cys171 residue within the intrinsically disordered region of Myc [16]. By binding to this unique residue, EN4 reduces stability of Myc and its obligate partner, Max, thus reducing the DNA binding ability of the Myc-Max complex and its capability to carry out proliferation and tumorigenesis activity in cancer cells. Another compound MYCi361 and its improved analogue, MYCi975, were developed to directly bind to and inhibit Myc activity [17]. These small molecules regulate Myc Thr58 phosphorylation and increase its proteasomal degradation, and therefore decrease Myc protein stability. Finally, L755507 is a recent preclinical chemical developed to target heterodimerization of Myc and Max to inhibit Myc transcriptional activation [18]. In that study, Singh et al. combined CADD approaches and experimental validation to design a new small molecule that disrupts the c-Myc-Max heterodimer, thus restricting growth of diverse Myc-expressing cells.

Given the high structural and functional similarity between the c-Myc and N-Myc oncoproteins [19,20], we used our previously-developed chemical series targeting Myc-Max/DBD complex as templates for the further development of novel N-Myc specific inhibitors [21,22]. We subsequently designed the next generation of compounds through synergetic use of structure-based screening, modeling, docking, medicinal chemistry efforts and experimental evaluation. Through this work we have identified compound VPC-70619, which blocks the N-Myc-Max heterocomplex from binding to DNA E-boxes and demonstrated strong inhibition activity against N-Myc-dependent cell lines as well as high bioavailability in both oral and intraperitoneal administration.

## 2. Results

### 2.1. Large-Scale Structure-Based Similarity Search and Scaffold Tuning

Previously, we identified a potentially druggable pocket where the Myc bHLHLZ domain interacts with its homologous domain from the Max protein, forming a stable helical configuration which binds specifically to DNA E-boxes at enhancers and promoters of Myc target genes [21]. By screening molecules targeting this site, we identified the initial hit VPC-70063 as a potential lead to inhibit Myc-downstream effects. Using the ROCS program, we performed ligand-based similarity searches against the drug-like purchasable chemical space of the ZINC15 library with VPC-70063 and related hits as query molecules and identified compounds with distinct chemical frameworks, shown in Table 1.

**Figure 1:**
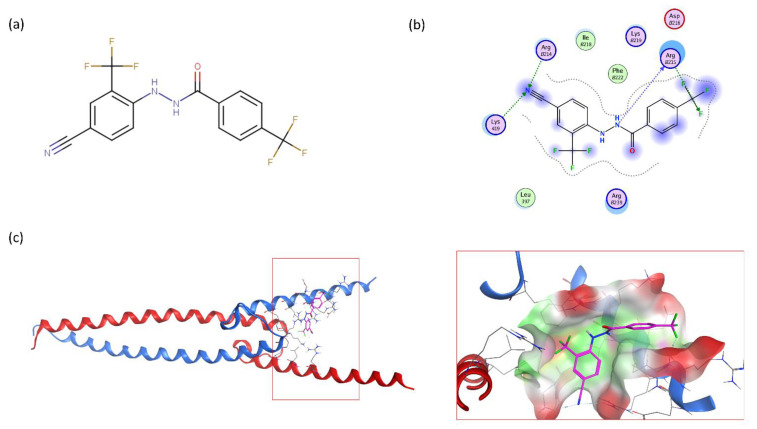
Proposed binding mode of 70551 into the N-Myc-Max DBD pocket. (**a**) 2D representation of the N-Myc specific lead, 70551. (**b**) The complete interaction map shows that 70551 interacts with three residues principally: Lys419 of N-Myc, and Arg214 and Arg215 of Max. Green dotted lines show interactions between the ligand and sidechain receptor; blue dotted lines show interactions between the ligand and the backbone receptor. Blue circles around the ligand’s atoms represent their level of solvent exposure. (**c**) 70551 in the proposed binding site of the homology model of N-Myc-Max DBD. A zoom of the site (right) shows that the compound has one strong H-bond with Max:Arg215 from 70551′s NH group in its hydrazide linker.

This similarity search yielded the hit compound VPC-70127, which is characterized by the presence of a 2-nitro-4-(trifluoromethyl)phenyl and a pyrazine connected via a hydrazide linker. Although VPC-70127 showed strong Myc inhibitory activity in our primary transcriptional assay, initial assessment demonstrated that VPC-70127 only weakly disrupts DNA binding to Myc-Max complex. Similarly, although VPC-70511 showed promising inhibitory activity in the transcription assay in LNCaP cells, further testing in a counter-screen using Myc-negative HO15.19, revealed that VPC-70511 had a highly cytotoxic profile. Thus, we pursued another hit, VPC-70551, which contains a similar hydrazide linker connecting two substituted phenyl rings together. The 2-nitro-4-(trifluoromethyl)phenyl was replaced by 4-cyano-2-(trifluoromethyl)phenyl and the pyrazine was replaced by a (trifluoromethyl)phenyl, as shown in Figure 1a.

To confirm the new scaffold’s specificity toward N-Myc, we performed a round of substructure searches using N’-phenylbenzohydrazide as a query against the ZINC15 database and selected 34 analogues for experimental validation (Table 2). We then observed that the absence of the 4-cyano group or 2-trifluoromethyl significantly reduced inhibition activity (VPC-70586 and -70611). Compounds with the cyano- and trifluoromethyl substitutions are significantly more active than their counterparts without (VPC-70593, -70596, -70599, and -70604). To determine the interactions responsible for the reported activity, we docked the active compounds to a homology model of N-Myc-Max. For our binding pose predictions, the same homology model of N-Myc-Max DBD as described in our previous publication was used [22].

The docking pose of VPC-70551 inside N-Myc-Max/DBD complex revealed that the compound forms an H-bond with the backbone of Max’s Asp215 through its hydrazide linker, and a pair of weaker H-bond with sidechain Lys419 and Arg214 of N-Myc through its cyano group. The 4-cyano-2-(trifluoromethyl)phenyl group is involved in numerous hydrophobic contacts with N-Myc residues Leu397, Phe401, and Lys419, and with Max residues Arg212, Arg215 Ile218, Lys219, Phe222, and Arg239. These contacts anchor VPC-70551 into its position in the DBD pocket (Figure 1c). A comparison between chemical structures of the analogues suggested that the N’-[4-Cyano-2-(trifluoromethyl)phenyl] group is the substituent responsible for the observed higher potency of VPC-70551 and its analogues in inhibiting Myc-Max transcriptional activity. Therefore, we utilized the N’-[4-cyano-2-(trifluoromethyl)phenyl]benzohydrazide scaffold of VPC-70551 to further explore improved hits.

### 2.2. Extensive N-Myc Specific SAR Exploration

**Figure 2:**
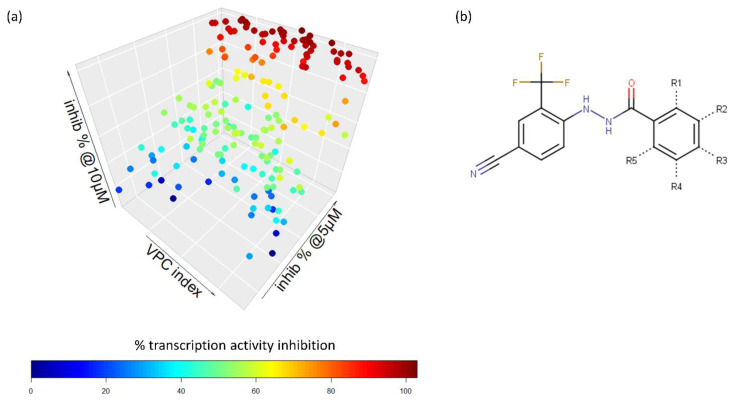
Screening results of all 181 N’-[4-cyano-2-(trifluoromethyl)phenyl]benzohydrazide derivatives. (**a**) The compounds were screened for inhibition of transcription activity in LNCaP-NMYC at 10 µM and 5 µM. Compounds of interest are coloured in dark red as they are active at both concentrations tested and were selected for viability assays. (**b**) Proposed N’-[4-cyano-2-(trifluoromethyl)phenyl]benzohydrazide scaffold used to identify 181 derivatives for further validation. Substitutions were made on positions R1 to R6 of the free phenyl ring. All experiments were performed with *N* = 3 replicates.

To determine the optimal substitutions on the free phenyl ring, we used the active scaffold for a second substructure search against the ZINC15 database to identify a set of 181 analogues for further validation (VPC-70617 to -70799, presented in Supplementary Table S1). Compounds were tested for their inhibition of a N-Myc-Max luciferase transcription activity assay at 5 and 10 µM Figure 2a [21]. Herein, we inspected all the proposed substitutions on the scaffold to discern the features required to construct a possible SAR model for inhibitors targeting N-Myc-Max/DBD complex (Figure 2b).

#### 2.2.1. Large, Extended, or Bulky Substitutions Are Detrimental to Anti-N-Myc Activity

The inhibitory potency of the analogues appeared to be sensitive to substitutions on the phenyl ring, especially at the R3 para-position. R3 substitution shifts ligands towards exposed surfaces of the N-Myc-Max pocket and can negatively affect the inhibitory activity. This negative effect is reported in bicyclic compounds and the majority of compounds with large or extended chemical groups that do not interact directly with the N-Myc-Max DBD (VPC-70620, 70627, 70629, 70634, 70637, 70643, 70645, 70657, 70660, 70661, 70667, 70668, 70669, 70670, 70671, 70677, 70678, 70681, 70683, 70687, 70689, 70690, 70691, 70718, 70724, 70727, 70731, 70735, 70737, 70745, 70751, 70752, 70753, 70763, 70771, 70781, 70785, 70786, 70791, and 70799). Bulky and large chemical groups were deemed unfavorable at the meta-substitutions (VPC-70649, 70748, 70795) as they would displace the scaffold completely from its proposed binding position and shift the compound to the solvent-exposed areas.

#### 2.2.2. Sulfonyl and Oxy-Linkers Did Not Improve Scaffold Potency

We then tested another series of substitutions with sulfonyl linkers at the R3-position. Combinations of different groups with the linker, such as methyl, ethyl, amine, or methylaniline with the sulfonyl linker, did not help their inhibitory activity (VPC-70623, 70641, 70655, 70665, 70692, 70694, 70697, 70717, 70764, 70789, 70790, 70793, and 70796)**.** Although the sulfonyl linkers are interacting with the Max protein, they cannot compensate for the unfavorable position of the ligands’ groups in the solvent-exposed area of the pocket. This lack of activity is observed in most compounds containing sulfur groups at para and meta positions (VPC-70630, 70676, 70682, 70688, 70729). Interestingly, three compounds (VPC-70658, 70733, and 70782) with sulfur or large linkers were active, and all three had fluorine groups.

We did not observe significant inhibitory activity in compounds with an oxy linker in their R3-extended group (VPC-70618, 70646, 70664, 70716, 70736, 70740, 70757, 70759, 70766, 70778, 70788), or compounds with para–OH or meta –OH substitutions (VPC-70626, 70744, 70749, 70758, 70762, 70773, 70774, 70779). We observed a few exceptions: VPC-70654, 70663, 70732 and 70792. Both VPC-70732 and 70792 had oxy linkers in their substitution group, while VPC-70663 had a R3-nitro group, and they still maintained good transcription inhibition activity.

#### 2.2.3. Halogenic Substitutions Are Beneficial for N-Myc Transcription Inhibition

Interestingly, most of the active compounds contained at least one regular halogenic substitution. Sixteen analogues contained an R3–Cl substitution combined with other groups at the meta- or ortho-positions. Combinations of R3–Cl with other halogens/halogenic group at the meta position demonstrated good inhibition at both concentrations tested (VPC-70621, 70622, 70705), while small halogens at ortho position abolished inhibitory activity (VPC-70624, 70632, 70642). At the meta positions, methyl groups (VPC-70617) or sulfonyl groups (VPC-70721) did not impart any inhibitory activity at 5 µM, while a methoxy group (VPC-70685) or cyano group (VPC-70662) recovered the activity. Interestingly, activity was maintained when R3–Cl was combined with ortho –OH groups (VPC-70633) or large halogenic groups (VPC-70706, 70797). We tried compounds with triple substitutions centered on the R3–Cl as well. Although compounds with ortho-halogens at R3–Cl were previously inactive, adding a third substitution recovered the inhibition activity (VPC-70719: R1–Cl, R3–Cl, R5–OH; VPC-70730: R1–Cl, R3–Cl, R4–F; VPC-70741: R1–F, R3–Cl, R4–F). The third substitution added often came from a compound considered active; thus, the third substitution could be inconsequential and only two substitution groups could be necessary for inhibitory activity.

Similar to the R3–Cl, we observed strong increase in inhibition activity when the R3-position is substituted for another halogen group such as –F, –Br, and –CF_3_ (–F: VPC-70647, 70659, 70679, 70680, 70686, 70696, 70715, 70726, 70734, 70746, 70767 and 70780; –Br: VPC-70635, 70743, 70775; –CF_3_: VPC-70693, 70704). The effects are diminished when the substitutions are combined with methyl-ending groups (VPC-70648, 70674, 70783). The activity is completely abolished when the groups are added at the ortho position (VPC-70642, 70711, 70713, 70798) or when combined with bulky chemical substitutions, such as sulfonamides (VPC-70728, 70747, 70760, 70770, 70777). The positioning of the substitutions seems to influence the inhibition activity as an R1–F, R3–F, and R4–F grouping nullified activity (VPC-70772), while an R1–F, R2–F, and R3–F did not (VPC-70715).

Based on the above observations, we wanted to determine if the activity is maintained or increased by halogen substitutions at the meta- or ortho-positions only. The activity of compounds with halogen substitutions at the R1 or R5 position (ortho) often declined considerably or was lost completely (ortho–F: VPC-70625, 70709, 70710, 70712, 70714, 70720, 70723, 70755; ortho–Cl: VPC-70631, 70638, 70701, 70707, 70708, 70725; ortho–Br: VPC-70636; ortho–I: VPC-70702). However, compounds such as VPC-70698, 70768, 70769, and 70776 have ortho-halogen substitutions and still showed high transcription inhibition activity. In general, compounds with meta-substitutions (meta–F: VPC-70639, 70644, 70739, 70742, 70794; meta–Cl: VPC-70640, 70675, 70695, 70700, 70761; meta–Br: VPC-70738, 70754; other halogens: VPC-70703, 70756) reported a better inhibition activity when compared to ortho-substitutions, although some had no activity (VPC-70684). If the substitution at the R3 position is a methyl-like group, the compounds had a lower transcription inhibition or no activity at all (meta–F: VPC-70765, 70784; meta–Cl: VPC-70628). We observed that too many halogenic groups hampered activity (VPC-70656). Compounds with methyl substitutions were often just inactive (VPC-70666), no matter which position they were: VPC-70651 with meta–CH_3_ had no activity; when replaced by meta–Cl, the compounds had 100% transcription inhibition activity at 5 µM. Similarly, for VPC-70672, removing the methyl group at the R3 position restored activity (VPC-70653).

Compounds with simultaneous halogen substitutions with at least one –Cl at the meta-positions of the scaffold are shown to be significantly more active. Combining –Cl with either –Cl, –Br, and –CF_3_ substitutions demonstrated very strong transcription inhibition (VPC-70650, 70652, and 70722). We observed that –Cl led to a strong effect that could compensate for other substitutions that were deemed inactive in other compounds. This is the case for VPC-70619 which substituted the meta –OH group of VPC-70673, for a –Cl, restoring the inhibitory activity at 5 µM.

Based on the above SAR considerations we have concluded that due to the presence of the strong electronegative –Cl group, mainly at the R3 position and to a lesser extent –F and –Br, there is a strong case to have at least one halogenic group or a functional group with significant electronegativity as a substituent on the free phenyl ring. We stipulated that halogenic groups might have a crucial role as a hydrophobic contact to anchor the ligand to Max. Consequently, we carried out further viability assays to determine whether the reported activity in -Cl compounds emerged from toxicity effects.

### 2.3. VPC-70619 Shows a Good Balance of Potency and Viability in Multiple N-Myc Expressing Cell Lines

We shortlisted 54 active compounds requiring further testing in cell viability assays to determine their inhibition effect on N-Myc-dependant cellular growth. The shortlisted compounds included all derivatives of the N’-[4-Cyano-2-(trifluoromethyl)phenyl]benzohydrazide scaffold which were active at both 10 µM and 5 µM in our LNCaP-NMYC transcription inhibition assays. The compounds are mainly characterized by substitutions at the para- and meta-positions, with at least one of the substituted groups being a halogenic or a strong electronegative group.

The 54 compounds were tested at 10 µM for their inhibition of the IMR32 human neuroblastoma cell line, in which the MYCN gene is amplified and actively expressed (Table 3). HO15.19 cell line (Myc-negative) was used in parallel as a compound specificity control. Compounds showing an inhibition of HO15.19 cell line greater than 20% at 10 µM were excluded for toxicity.

**Figure 3:**
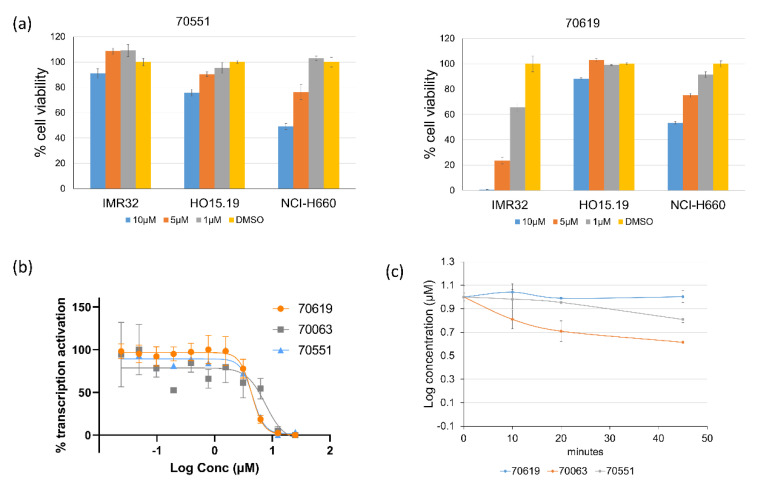
Inhibitory profile and viability of 70619 against N-Myc driven cell lines. (**a**) 70551 and 70619 were assessed in IMR32, NCI-H660 (N-Myc positive) and HO15.19 (N-Myc negative) at concentrations of 10 µM, 5 µM, and 1 µM. 70619 did not report any strong inhibitory effects in the counter-screen. All experiments were performed with *n* = 3 replicates. (**b**) Reported IC_50_ of 70063, 70551, and 70619 in the transcriptional assay following treatment for 1 day with increasing concentrations (0–25 µM). (**c**) Microsomal stability for compounds 70063, 70551, and 70619 were also determined. 70619 was shown to be highly stable with a T_1/2_ of 2310 min while we reported T_1/2_ of 141 and 69 min for 70551 and 70063, respectively.

We reported 13 compounds with minimal toxicity effects in the Myc-negative HO15.19 cell lines. From the 13 compounds, only one compound (VPC-70619) was effective in the IMR32 cell lines with an inhibition higher than 50%. At 10 µM, VPC-70619 had an inhibition of 99.4% in IMR32 cells while having a minimal effect of 14% in HO15.19 cells, thus showing potential selective activity against N-Myc expressing cells. We reported that VPC-70619 shows good inhibition of NEPC in the in vitro model NCI-H660 cell line. Furthermore, VPC-70619 had an IC_50_ of 4.3 µM in the luciferase transcription inhibition assay, and presented a stable microsomal half-life of 2310 min, compared to 3 min for 10074-G5 (Figure 3).

### 2.4. VPC-70619 Interaction with N-Myc-Max Complex Interferes with Its Binding to the DNA

**Figure 4:**
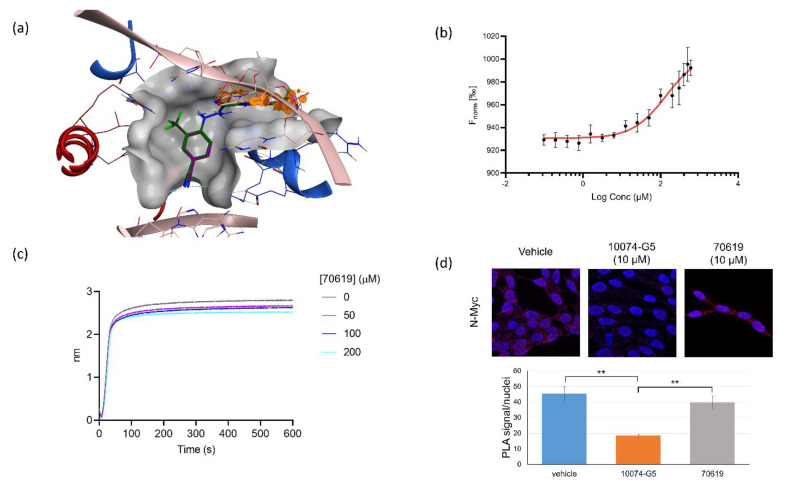
70619 blocks DNA binding to N-Myc-Max without disrupting the heterodimer. (**a**) Docking poses of 70619 and 70551 in the proposed N-Myc-Max DBD pocket. 70619 improves on the previous interactions established by 70551 and is shown to clash extensively with DNA E-boxes. Red: Myc; blue: Max; dark green: 70619; purple: 70551; pink: consensus DNA E-box. (**b**). Dose-dependent direct interaction of 70619 with the purified N-Myc-Max complex, measured by microscale thermophoresis (MST); 70619 is shown to bind weakly to the N-Myc-Max complex, due to the nature of the proposed binding pocket. MST assays were performed with *N* = 5 replicates. (**c**) Inhibition of DNA binding to Myc-Max by 70619 was quantified by biolayer interferometry (BLI) at concentrations of 0 µM, 50 µM, 100 µM, and 200 µM; 70619 was able to disrupt DNA binding at 100 µM. All experiments were performed with *N* = 3 replicates. (**d**) Proximity ligation assay comparing N-Myc-Max interactions treated without any inhibitor, with 10 µM of 10074-G5, and with 10 µM of 70619 in LNCaP-NMYC cells for 72 h. Interacting Myc-Max heterodimers are shown as red dots, and stained nuclei are shown in dark blue. Interacting heterodimers are quantified by dots per nuclei; 70619 does not disrupt Myc-Max complexes, as there is a significant decrease in the number of interactions in the 10074-G5 treated cells compared to either vehicle or 70619 (*p* < 0.01). No significant difference was observed between vehicle cells and cells treated with 70619.

Based on a promising activity profile of VPC-70619, we studied its binding pose in greater details. We observed that VPC-70619 has a similar docking profile to VPC-70551 thanks to their common shared scaffold. The compound maintained most of the same interactions, including engagement to the side chains of Lys419 of N-Myc. Additionally, side chains Lys219, Asp216, Arg212, and Arg215 act as clamps to anchor the phenyl ring into a stable conformation in the pocket through hydrophobic interactions (Figure 4). Although the cyano group itself does not interact with Myc-Max, the group is important to maintain for microsomal stability, and clashes directly with the DNA E-box.

As seen in the docking poses, the 3-chloro-4-cyano benzohydrazide portion of VPC-70619 overlaps significantly with the DNA backbone (Figure 4a). These groups are in direct contact with DNA E-box recognition sequences. More specifically, the chloro and cyano groups are colliding significantly with the phosphate group of DC110 (third position in the E-box recognition sequence), disrupting its H-bond with Max side chains Arg215 and Arg212. The carbonyl oxygen of the hydrazide linker sterically clashes with the phosphate group DA109 (2nd position), disrupting it from interacting with Arg239 of Myc and Lys219 of Max. The proposed binding mode of VPC-70619 suggests that the compound can compete with DNA binding to disrupt N-Myc-Max-DNA interaction.

We first performed a microscale thermophoresis (MST) assay to validate the direct binding of VPC-70619 to the recombinant N-Myc-Max complex and to calculate the binding affinity. We evaluated the effect of increasing concentrations of 70619 on the movement of a fluorescent labelled N-Myc-Max complex through a temperature gradient induced by an infrared laser. VPC-70619 demonstrated a dose-dependent shift of thermophoresis; however, due to the limited solubility of VPC-70619 in the assay buffer, we could only estimate a dissociation constant above 130 μM (Figure 4b).

To confirm that 70619 could disrupt the binding of N-Myc-Max DBD to DNA, we performed a bio-layer interferometry (BLI) assay where we immobilized biotinylated E-box sequence on streptavidin sensors, and we evaluated recombinant N-Myc-Max binding to the DNA in the presence of increasing concentrations of VPC-70619. We found that the compound was effective at blocking N-Myc-Max binding to DNA in a dose-dependent manner (Figure 4c). We finally tested VPC-70619 in a proximity-ligation assay (PLA) to determine whether the compound was interfering with the N-Myc-Max heterodimer, and demonstrated that the chemical does not disrupt the N-Myc-Max heterodimer formation in LNCaP cells. We observed a similar number of interactions when comparing the ratio of signal per nuclei in the presence or absence of the compound. Comparatively, in the presence of the Myc-Max dimer inhibitor 10074-G5, the number of interactions is reduced, indicating a decrease in N-Myc-Max heterodimer formation (Figure 4d).

### 2.5. Pharmacokinetic Study of VPC-70619 Reveals Good Intraperitoneal and Peroral Tolerance

**Figure 5:**
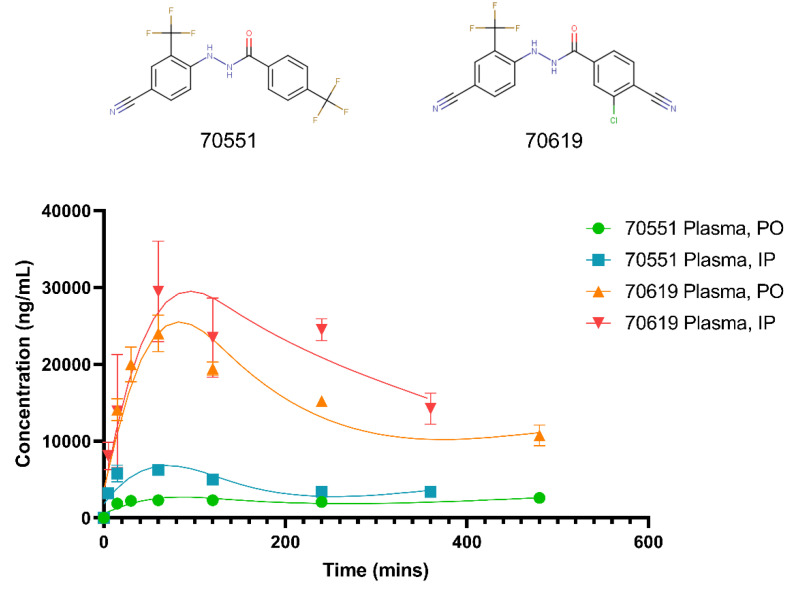
Concentration-time curves for 70551 and 70619 in male Balb/c mice following PO, and IP dosing. PK studies of 70619 and its parental compound, 70551, were carried out to determine the pharmacokinetic characteristics of both compounds following peroral (PO) and intraperitoneal (IP) administrations. Levels of the compounds were determined by LC-MS/MS in blood plasma over time after a single dose; 70619 is deemed to have an improved profile with higher bioavailability for both PO and IP administrations.

In order to characterize VPC-70619 as a drug candidate against N-myc, its pharmacokinetic (PK) characteristics were evaluated in Balb/c mice following intraperitoneal (IP) and peroral (PO) administrations. As presented in Figure 5, we determined the PK properties of VPC-70619 and compared it to the parental molecule, VPC-70551. For the latter, the peak plasma concentrations (T_max_) occurred at 480 min for PO and 60 min for IP, with peak concentrations (C_max_) of 2600 ng/mL and 6220 ng/mL, respectively, following administration in 75:25 propylene glycol (PG)/poly-(ethylene glycol) 400 (PEG) formulation at 10 mg/kg dosing. Plasma concentrations of 70551 decreased slowly, with a half-life (T_1/2_) of 332 min for IP administration. The clearance of 70551 for PO delivery was extremely slow, and we could not determine the T_1/2_ in the experimental conditions used for this assay. No obvious adverse effects of 70551 were observed in the tested animals. Regarding 70619, it presented much higher bioavailability, with a C_max_ of 29,500 ng/mL and 24,000 ng/mL for IP and PO, respectively, following administration in a 20:80 Kolliphor EL formulation. Plasma concentrations decreased slowly, with a reported terminal half-life of 330 min for IP, while PO had a T_1/2_ of 427 min. No signs of toxicity were observed in any control or treated animals. Therefore, VPC-70619 was deemed to have appropriate efficacy in vivo, and a better pharmacokinetic profile due to higher bioavailability for both oral and intraperitoneal administration.

## 3. Discussion

While N-Myc is not significantly expressed in adult tissues, it was reported to be amplified and overexpressed in NEPC tumors [23]. The presence of N-Myc alteration and deregulation drives tumor proliferation and progression, leading to a low survival rate in patients affected by NEPC [23]. Significant efforts were carried out to indirectly inhibit Myc-driven tumors by either interfering with the expression of Myc at the DNA (gene transcription), RNA (mRNA translation) and protein level (stability), or by targeting Myc genes with synthetic lethality [24]. Successful inhibition of the N-Myc-Max complex formation has shown to be an effective therapeutic strategy [25]; however, there are currently no clinically-approved compounds that were designed to interact directly with the N-Myc-Max DBD to treat PCa. Targeting N-Myc directly could be advantageous to avoid the likelihood of resistance emerging due to redundancy and parallelism in the oncogenic pathways [26,27]. Therefore, successful inhibition of “undruggable” and yet major oncoproteins like Myc would constitute a critical step forward for the plausibility of modulating difficult transcription factor proteins, opening the door for the possibility to develop therapeutics for a wider range of cancers. Consequently, integrating novel CADD approaches would play an important role in targeting novel sites without classical properties, such as those with unusual and convex pocket shapes or transient protein–protein interactions [28].

In our previous studies, we described a novel anti-Myc compound series targeting the proposed Myc-Max heterodimer DBD pocket [21], and reported the development of a dual N-Myc and AURKA inhibitor using the currently identified scaffold [22]. Due to toxicity effects and microsomal instability in our initial compound series, we sought to optimize our scaffold’s N-Myc specificity to reduce cytotoxicity, and explored ways to increase microsomal stability of chemicals. For our CADD experiments, we utilized the same homology model of the N-Myc-Max DBD complex as previously described, and established an active scaffold based on similarity and substructure searches. The large-scale structure-based similarity searches allowed us to identify the N’-phenylbenzohydrazide scaffold of VPC-70551 as a potential starting point for optimized N-Myc inhibitors. We validated a refined scaffold through a second structural similarity search. This fine-tuned search confirmed that the addition of a 4-cyano-2-(trifluoromethyl) substitution to the phenylbenzohydrazide enhanced the inhibitory activity.

To improve the potency in the series, we explored the enhanced N-Myc scaffold by searching for new compounds with substitutions on the empty phenyl ring. Extensive SAR analysis revealed that successful inhibition relies on the presence of an electronegative group such as halogens at the para- or meta position of the phenyl ring. The halogen substitutions, combined with another small group, maintained and enhanced activity or specificity. Docking simulations show that the halogens at the para or meta positions interact with the N-Myc-Max DBD site through a network of hydrophobic contacts, while halogen substitutions at the ortho position do not impart any activity as they are not able to interact with the pocket or are displaced by the compound completely when bound. Similarly, large, extended, and bulky substitution groups are detrimental to the scaffold’s anti-N-Myc activity, as they often displace the compound from the pocket in our docking simulations. We tested multiple series of ligands, including sulfonyl, methyl, ethyl, and oxy linkers, and the majority of them did not return significant improvement over the parental compound, VPC-70551. We stipulate that they could not enhance the proposed interaction network that was already in place, or could not compensate for the unfavorable solvation of the ligands’ exposed groups. Our binding and SAR model provides valuable insight into the proposed N-Myc-Max DBD pocket, and suggests that active compounds must bind tightly to both N-Myc and Max and minimize solvent-exposed groups.

Using cell-based screening, we could exclude molecules presenting off-target effects or toxicity. We identified a potent compound, VPC-70619, characterized by the same 4-cyano-2-(trifluoromethyl)phenyl-benzohydrazide scaffold which anchors the compound into the hydrophobic core of the N-Myc-Max DBD pocket, as well as a 2-chlorobenzonitrile group which clashes with the DNA E-box binding to N-Myc-Max. Interestingly, 70619 has 15-fold increased microsomal stability, with a half-life of 2310 min compared to 140 min for VPC-70551. The lead compound, 70619, inhibits cell proliferation levels in the N-Myc-driven NCI-H660, with a low micromolar IC_50_ of 7.0 μM. Importantly, 70619 was effective at inhibiting growth in N-Myc-specific cells (IMR32 and NCI-H660) while presenting minimal cytotoxicity in the Myc-negative HO15.19 cell lines. The compound was shown to bind to recombinant N-Myc-Max complex and to block DNA binding without disrupting the N-Myc-Max heterodimer itself. However, VPC-70619 presented somewhat weak affinity to N-Myc-Max; this might be due to the nature of the proposed pocket, and thus further optimization of the molecule is needed to ensure the success of our structural determination of the N-Myc-Max interaction with the identified inhibitors. Nonetheless, we propose that 70619′s mode of action in inhibiting N-myc cell proliferation is via the blocking of the N-Myc-Max heterocomplex from binding to target genes.

Based on the proposed SAR model, including a halogenic group in our series is highly beneficial to the reported anti-N-Myc activity. As previously highlighted, their inclusion as hydrophobic moieties could therefore be important as molecular interaction with weak to medium strength [29], specifically with Max’s sidechain in the proposed pocket. Therefore, active compounds in the series could be relying primarily on hydrophobic contacts to interact with N-Myc-Max, and the addition of H-bonds could be only slightly beneficial to the overall interaction network. Critically, the presence of a halogenic group seemed to be significantly involved in disrupting DNA binding to N-Myc-Max DBD. For our lead compound, VPC-70619, both halogenic and nitrile groups interfere with the DNA at multiple positions. We observed that inclusion of nitriles is important for overall microsomal stability, in accordance with what has been proposed elsewhere [30]. Consequently, it will be important to fine-tune the balance between the potency and cytotoxicity of the reported anti-N-Myc compound series for better optimization.

Thus, we propose compound VPC-70619 as a single agent that could be effective with tolerable toxic effects within a specific therapeutic window, or as a chemotherapy agent which can be part of a combined oncogenic treatment regimen [31,32]. Pharmacokinetic studies revealed that VPC-70619 has high bioavailability by the intraperitoneal and, importantly, oral administration, which both have distinct advantages as alternate routes over intravenous administration, most notably by avoiding the first-pass effect of hepatic metabolism [33]. The pharmacokinetic profile of VPC-70619 is a significant improvement over our previous hit compounds, and represents an important step forward. Significant efforts should now be devoted to improving its viability profile. The reported scaffold of VPC-70619 has successfully guided the CADD pipeline and our hit selection. It provided new insight into the proposed N-Myc-Max DBD pocket and could represent possible starts for therapeutic development of a direct anti-N-Myc drug candidate with high potency and selectivity. This new class of N-Myc inhibitor demonstrates that interfering directly with N-Myc-Max’s ability to interact with DNA E-boxes could be a viable mechanism for the design of a novel type of anti-cancer drugs to treat lethal NEPC.

## 4. Materials and Methods

### 4.1. Computational Methods

#### 4.1.1. Chemical Similarity Searches

To identify potentially improved derivatives, high-similarity 3D structural searches were performed on the ZINC15 database [34] using the ROCS program from OpenEye [35]. Omega2 from OpenEye (Santa Fe, NM, USA) was used to generate the different conformers of each searchable compound [36], and 2D substructure-based searches were performed against the ZINC15 database. Hit compounds from similarity and substructure searches were ranked per their Tanimoto score. Specialized small molecules were received from ENAMINE-REAL from our similarity searches, and custom-made molecules received from Life Chemicals were prepared for docking simulations.

#### 4.1.2. Protein and Ligand Preparation

The Protein Preparation Wizard tool within Maestro 9.3 from Schrödinger LLC (New York, NY, USA) [37] was used to prepare the N-Myc-Max DNA-binding pocket homology model and to add adequate hydrogen atoms to the proteins. The protein structures were then submitted to energy minimization using the OPLS3e forcefield until RMSD reached convergence at 0.3 Å, relative to the starting geometry [38].

#### 4.1.3. Docking

Docking analysis of all compounds obtained from similarity searches or medicinal chemistry synthesis was performed with the Glide docking program from Schrödinger LLC [39]. A docking grid was generated by setting a 20 Å box centered in the suggested binding region of the N-Myc-Max DBD structure. The compounds were docked into the N-Myc-Max DBD pocket using Glide SP (Standard Precision) mode. The docking poses were then scored and ranked accordingly.

### 4.2. In Vitro Evaluation of Hit Compounds

#### 4.2.1. Cell Lines

LNCaP-NMYC cells were a generous gift from David Rickman at Weill Cornell Medicine (New York, NY, USA), and were cultured in RPMI supplemented with 10% fetal bovine serum (FBS). HO15.19 cells cultured in DMEM supplemented with 10% FBS were a generous gift from John Sidivy at Brown University (Providence, RI, USA). NCI-H660 cells were purchased from the American Type Culture Collection (Manassas, VA, USA) and cultured in RPMI supplemented 5% FBS, 1% Insulin-Transferrin-Selenium (Thermo Fisher, 41400-045) (Waltham, MA, USA), 10nM b-estradiol (Sigma, E8875) (St. Louis, MO, USA), 10 nM hydrocortisone (Sigma H0888) and 1% matrigel. IMR32 cells were purchased from ATCC and were cultured in Eagle’s Minimum Essential Medium (EMEM) supplemented with 10% FBS.

#### 4.2.2. Myc Transcription Assay

LNCaP-NMYC cells were transfected using TransIT-2020 transfection reagents per the manufacturer’s protocol. Cells were plated at a density of 10,000 cells per well of a 96-well plate. Following 24 h treatment with 5 µM or 10 µM of the compounds, the Myc reporter activity was measured using the Cignal Myc Reporter Assay Kit from Qiagen (#336841) (Hilden, Germany) per the manufacturer’s instructions.

#### 4.2.3. Cell Viability

Viability of N-Myc positive (IMR32and NCI-H660) and negative (HO15.19) cells were determined using the CellTiter-Glo Luminescent Cell Viability Assay (Promega cat. G7570) (Madison, WI, USA). Cells were seeded into a 96-well white plate with a clear bottom: 10,000 cells per well for the IMR32 cells cultured in EMEM supplemented with 10% FBS, 2000 HO15.19 cells per well in Eagle’s minimum essential medium (EMEM) supplemented with 10% FBS, and 2000 cells per well for the NCI-H660 neuroendocrine cells cultured in RPMI supplemented 5% FBS, 1% Insulin-Transferrin-Selenium (Thermo Fisher, 41400-045), 10nM b-estradiol (Sigma, E8875), 10nM hydrocortisone (Sigma H0888) and 1% matrigel. Following 24 h incubation, the cells were treated with serial dilutions of the derivatives along with the respective parental compound starting at 25 μM for 72 h. Cell Titer Glo was added to each well at a ratio of 1:1 and incubated on a shaker at RT for 2 min. Luminescence and IC_50_ was measured with the TECAN InfiniteM200 plate reader (Männedorf, Switzerland).

#### 4.2.4. Protein Purification

The bHLHLZ domain of N-Myc (amino acids 309–394) and Max (amino acids 12–93) protein sequences were cloned into pETDuet with an N-terminal 6×His. pETDuet-His-NMyc-Max was transformed and expressed in E. coli BL21 and induced with 0.5 mM isopropyl-β-D-thiogalactopyranoside (IPTG) at 16 °C overnight. The cultures were lysed by sonication and combined for purification by immobilized metal ion chromatography (IMAC) with nickel-nitrilotriacetic acid (Ni-NTA) resin. The bound proteins were eluted from the column with 20 mM Tris, pH = 8, 500 mM NaCl, 5% glycerol, 300 mM imidazole, 5 mM βMe, and 0.1 mM PMSF. Size exclusion chromatography with Superdex 75 was used to increase the purity of the N-Myc-Max protein sample. The purified protein complex was used in subsequent BLI and MST experiments.

#### 4.2.5. Biolayer Interferometry Assay

Biotinylated-duplex DNA (Forward: 5′ Biotin-TGAAGCAGACCACGTGGTCGTCTTCA, Reverse: TGAAGACGACCACGTGGTCTGCTTCA) was bound to the super-streptavidin (SSA) ForteBio Sensor in the assay buffer [20 mM Tris pH = 8, 150 mM NaCl, 5% glycerol, and 0.2 mM TCEP]. The protein solution was mixed with the serially diluted compound (70619) to a final concentration of 0.1 mg/mL of protein and 200 μM, 100 μM, and 50 μM for the compound. Sample containing N-Myc-Max protein alone was used as a control.

#### 4.2.6. Proximity Ligation Assay (PLA)

LNCaP-NMYC cells were seeded on coverslips at a density of 10^5^ cells per well of a 6-well plate. After incubation for 24 h the cells were treated with 10 μM of either 10074-G5 or 70619 for 72 h. The coverslips were fixed with methanol:acetone (3:1) and incubated with primary antibodies n-Myc Antibody (NMYC-1) (Novus Biologicals NB200-109) (Littleton, CO, USA) and MAX Antibody (C-17) (Santa Cruz sc-197) (Dallas, TX, USA) overnight at 4 °C. PLA was conducted using the Duolink In Situ Detection Reagents Red kit (Sigma DUO92008) according to the manufacturer’s protocol. The coverslips were then mounted on slides with VECTASHIELD mounting media with DAPI, and images were taken on ZEISS 780 confocal microscope at 40× magnification.

#### 4.2.7. Pharmacokinetics Studies

Bienta Enamine Biology Services (Kyiv, Ukraine) conducted PK studies according to the Enamine PK study protocols and Institutional Animal Care and Use Guidelines. The PK studies were reviewed and approved by the Bienta Animal Care and Use Committee (BACUC) under the project identification codes PK-VPC-112618 (approved 17 December 2018) and PK-VPC-101119 (approved 21 October 2019), for VPC-70551 and VPC-70916, respectively. Evaluation of 70619 and 70551 was conducted in male Balb/c mice through intraperitoneal (IP) and peroral (PO) administration. Measurements were taken at six time points: 5, 15, 60, 120, 240, and 360 min for IP; 15, 30, 60, 120, 240, and 480 min for PO. Four mice were included in each treatment group for each time point; one mouse each for IP and PO was included as a control. Initial target administration for both IP and PO was 10 mg/kg dose level, 2 mg/mL dose concentration, and 5 mL/kg dose volume. Concentration of the compounds was evaluated in the plasma samples using an LC-MS/MS system.

#### 4.2.8. Microsomal Stability Assay

A microsomal stability assay was performed using the Corning Gentest NADPH Regenerating System (Corning, #451220/41200) (Corning, NY, USA). Mouse liver microsomes (MLM) (Xenotech, M1000) (Kansas City, KS, USA) and compounds were diluted to a final concentration of 0.15 mg/mL and 1 μM respectively. These were prepared into a 500 μL solution with 100 mM potassium phosphate buffer (pH = 7.4). Imipramine was used as a control. NADPH solutions A and B were mixed in a 5:1 ratio, and 24 μL of the NADPH was added to each of the MLM/compound samples at 30 s intervals. At time points 10, 20, and 45 min, 100 μL of reaction was added to the stopping solution. Samples were centrifuged at 18,000× *g* and incubated at 4 °C for 10 min. Samples were then loaded onto a LC-MS 96-well plate for analysis.

#### 4.2.9. Thermophoresis (MST)

An MST assay was conducted to determine the binding affinity of 70619 to the purified N-Myc-Max protein complex. N-Myc-Max protein samples were labeled with the red fluorescent dye NT647 using the Monolith NT Protein labeling kit RED-NHS amine-reactive (NanoTemper Technologies, München, Germany); 70619 was serial diluted with 100% DMSO and mixed with the labeled fluorescent N-Myc-Max (final concentration of protein: 5 nM) in the assay reaction buffer (20 mM Tris, pH = 8, 100 mM NaCl, 0.2 mM TCEP, 0.1 mM PMSF, 5% glycerol) starting at 600 μM, with a final concentration of DMSO of 5%. The protein/inhibitor solution was mixed and incubated at room temperature in the dark for 5 min before being filled into the capillaries. MST assays were performed with 20% LED/excitation power and medium MST power using premium capillaries for Monolith NT.115. Analysis of the data for Kd estimation was calculated with the MO Affinity Analysis software from Nanotemper (München, Germany).

## 5. Conclusions

In this study, we determined that N’-[4-Cyano-2-(trifluoromethyl)phenyl]benzohydrazide derivatives developed by CADD modelling synergized with experimental validation are potent and selective N-Myc inhibitors. We further reported that the previously-identified binding site on the N-Myc-Max/DBD complex represents a viable drug target for inhibition with small molecules. The lead compound VPC-70619, identified here from multiple rounds of structural similarity searches and molecular docking, demonstrated strong anti-proliferative activity against a neuroendocrine cell line. While we identified a potential therapeutic window that could situate VPC-70619 as a potential candidate in anti-Myc treatments, supplemental optimization studies will be required to determine its full potential for future clinical applications. We identified novel derivatives through extensive SAR studies of structural analogues, providing valuable insight into N-Myc-Max DBD site topology. Finally, we have proposed that interfering directly with the ability of N-Myc-Max to interact with DNA E-boxes could be a viable mechanism for the design of potent small molecules to treat NEPC patients.

## 6. Patents

All compounds reported in this manuscript can be made available to other researchers after standard Material Transfer Agreement (MTA) implementation with the University of British Columbia.

U.S. Patent Application Number: 17/250,810

Filed 5 March 2021 based on PCT Application No. PCT/CA2019/051243

Title: MYC-MAX INHIBITOR COMPOUND THERAPEUTICS FOR CANCER TREATMENT, METHODS AND USES ASSOCIATED THEREWITH

Inventors: TCHERKASSOV, A.; RENNIE, P. S.; BAN, F.; LEBLANC, E. J. J.; CARABET, L. A.; LALLOUS, N.; SINGH K.;, MORIN, H.; and TON, A.

## Figures and Tables

**Table 1:** Initial hit compounds from ROCS similarity search using VPC-70063 and related hits as templates.

ID	Structure	IC_50_ (µM)	LNCaP-NMYC % Inhibition (25 µM)
70127	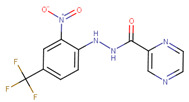	1	106
70215	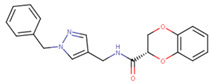	20	92
70223	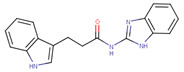	10	103
70314	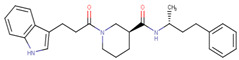	15	93
70381	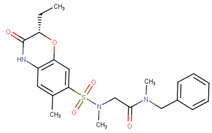	20	74
70388	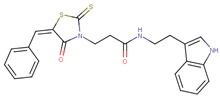	20	77
70390	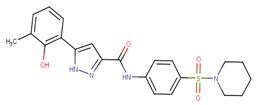	9	97
70465	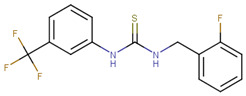	10	92
70495	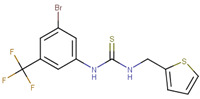	5	100
70511	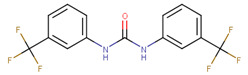	2	102
70551	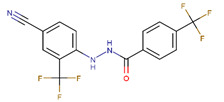	4	98

Compounds were tested in a transcriptional assay in LNCaP cells overexpressing N-Myc to validate Myc activity.

**Table 2:** Analogues of 70551′s N’-phenylbenzohydrazide active scaffold.

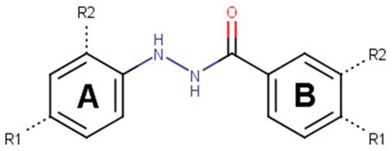							
ID	Ring A		Ring B		% Transcript Inhibition (10 µM)	% Transcript Inhibition (5 µM)	IC_50_ (µM)
	R1	R2	R1	R2			
70582	C≡N	CF_3_	C≡CH	H	84	44	8
70583	CF_3_	H	CF_3_	H	28	8	>20
70584	CF_3_	H	C≡N	H	30	34	>20
70585	H	F	CF_3_	H	24	16	>20
70586	C≡N	H	CF_3_	H	34	28	>20
70587	C≡N	H	C≡N	H	14	−58	
70588	C≡N	CF_3_	O–CH–F_2_	H	46	32	
70589	C≡N	CF_3_	I	H	94	64	3
70590	C≡N	CF_3_	CH=CH2	H	62	38	15
70591	C≡N	CF_3_	F	H	64	18	25
70592	C≡N	CF_3_	CO–CH2	H	8	4	
70593	C≡N	CF_3_	C≡N	H	70	−26	12
70594	C≡N	CF_3_	C–CH_3_–CH_3_-CN	H	10	22	
70595	C≡N	CF_3_	CH–CH_3_–CH_3_	H	70	30	12
70596	C≡N	CF_3_	H	CF_3_	94	96	2
70597	C≡N	CF_3_	CF_3_	H	82	26	12
70598	C≡N	CF_3_	CH–CF_2_	H	60	−4	15
70599	C≡N	CF_3_	Cl	H	78	96	3
70600	C≡N	CF_3_	CH_3_	H	10	−22	
70601	C≡N	CF_3_	CO–NH_2_	H	−12	16	
70602	C≡N	CF_3_	Br	H	48	12	
70603	C≡N	CF_3_	H	H	−12	−30	>20
70604	C≡N	CF_3_	C–C_3_H_6_	H	78	24	8
70605	C≡N	CF_3_	N–C_2_H_6_	H	26	−74	
70606	H	CF_3_	C–CH_3_–F_2_	H	−10	0	
70607	C≡N	H	C–CH_3_–F_2_	H	−8	−20	
70608	C≡N	CF_3_	C–CH_3_–F_2_	H	38	−14	
70609	H	CF_3_	F	H	12	20	
70610	H	CF_3_	C–C_3_H_6_	H	36	24	
70611	H	CF_3_	CF_3_	H	4	−28	>20

Compounds were tested in a transcriptional assay in LNCaP cells overexpressing N-Myc to validate Myc activity.

**Table 3:** Set tested for viability in IMR32 and HO15.19.

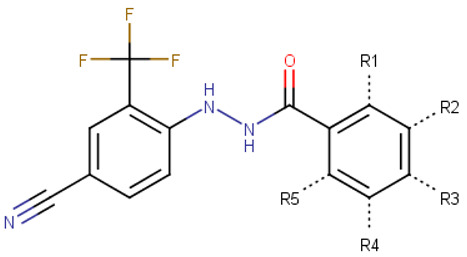							
ID	Substitutions					% Inhibition in IMR32 (10 µM)	% Inhibition in HO15.19 (10 µM)
	R1	R2	R3	R4	R5		
70619	H	Cl	C≡N	H	H	99.4	14.1
70621	H	F	Cl	H	H	98.8	68.6
70622	H	Br	Cl	H	H	99.4	36.4
70633	OH	H	Cl	H	H	93.6	77.0
70635	H	Cl	Br	H	H	7.5	11.8
70639	H	F	H	F	H	60.2	66.1
70640	OH	H	H	Cl	H	93.5	76.7
70644	H	Cl	H	F	H	88.3	69.5
70647	H	CF3	F	H	H	88.6	70.9
70650	H	Br	H	Cl	H	92.1	80.2
70652	H	Cl	H	Cl	H	92.5	80.7
70654	H	H	CO–O–C–C–C_3_H_6_	H	H	17.0	13.9
70658	H	H	S-CF_3_	H	H	46.9	57.2
70659	Cl	H	F	F	H	31.0	31.5
70662	H	C≡N	Cl	H	H	11.0	9.9
70663	H	H	NO_2_	H	H	28.9	7.9
70672	H	C≡N	H	H	H	18.0	8.1
70675	H	Cl	H	CH_3_	H	65.4	65.9
70679	H	CH_3_	F	CH_3_	H	1.1	7.3
70680	H	Cl	F	H	H	52.9	64.0
70682	H	H	S–CF_2_	H	H	11.0	22.9
70685	H	O–CH_3_	Cl	H	H	24.7	15.2
70686	H	F	F	F	H	82.2	75.8
70693	H	F	CF_3_	H	H	22.4	0.2
70695	H	Cl	NH_2_	Cl	H	77.1	41.2
70696	H	C≡N	F	H	H	15.1	13.2
70698	Cl	H	H	C≡N	H	35.4	27.3
70700	H	O–CH_3_	H	Cl	H	60.5	42.0
70703	H	I	H	H	H	18.5	13.5
70704	Cl	H	CF_3_	H	H	10.6	13.6
70705	H	Cl	CF_3_	H	H	60.7	61.8
70706	CF_3_	H	Cl	H	H	54.3	65.6
70715	F	F	F	H	H	14.4	39.9
70719	OH	H	Cl	H	Cl	95.0	76.5
70722	H	CF3	H	Cl	H	94.7	79.3
70726	H	I	F	H	H	75.6	66.2
70730	Cl	H	Cl	F	H	87.5	77.1
70732	H	H	O–phenyl–CO–CH_3_	H	H	98.9	98.9
70733	H	H	C–C_2_H_6_–CF_3_	H	H	57.4	57.8
70734	H	Br	F	H	H	86.5	77.8
70739	H	Br	H	F	H	87.2	75.8
70741	F	H	Cl	F	H	16.4	14.5
70742	H	F	I	H	H	26.1	71.8
70743	H	F	Br	H	H	38.7	74.9
70756	H	CF_3_	H	CF_3_	H	95.7	84.1
70761	H	NO_2_	H	Cl	H	93.3	81.0
70767	H	F	Cl	F	H	31.6	40.3
70768	F	Br	H	Cl	H	35.3	39.8
70769	H	CF_3_	H	H	F	32.2	28.2
70775	H	F	Br	Cl	H	62.4	65.1
70776	Br	F	H	Cl	H	27.4	26.1
70792	H	O–CF_2_	H	O–CF_2_	H	84.9	79.5
70794	H	CF_3_	H	F	H	89.2	80.7
70797	OH	H	CF_3_	H	H	24.7	34.2

## Data Availability

Not applicable.

## Supplementary Materials

The following supporting information can be downloaded at: https://www.mdpi.com/article/10.3390/ijms23052588/s1.
